# Characterization of PANoptosis-related genes in Crohn’s disease by integrated bioinformatics, machine learning and experiments

**DOI:** 10.1038/s41598-024-62259-w

**Published:** 2024-05-22

**Authors:** Yang Yang, Alphonse Houssou Hounye, Yiqian Chen, Zhuqing Liu, Guanzhong Shi, Ying Xiao

**Affiliations:** 1 Department of Gastroenterology, Xiangya Hospital, Central South University, Changsha, Hunan China; 2 Hunan International Scientific and Technological Cooperation Base of Artificial Intelligence Computer Aided Diagnosis and Treatment for Digestive Disease, Changsha, Hunan China; 3 National Clinical Research Center for Geriatric Disorders, Xiangya Hospital, Central South University, Changsha, Hunan China; 4 Xiangya School of Medicine, Central South University, Changsha, Hunan China; 5 School of Mathematics and Statistics, Central South University, Changsha, 410008 China

**Keywords:** PANoptosis, Crohn’s disease, Bioinformatics, Machine learning, Regulatory networks, Crohn's disease, Crohn's disease, Computational biology and bioinformatics

## Abstract

Currently, the biological understanding of Crohn’s disease (CD) remains limited. PANoptosis is a revolutionary form of cell death reported to participate in numerous diseases, including CD. In our study, we aimed to uncover the roles of PANoptosis in CD. Differentially expressed PANoptosis-related genes (DE-PRGs) were identified by overlapping PANoptosis-related genes and differentially expressed genes between CD and normal samples in a combined microarray dataset. Three machine learning algorithms were adopted to detect hub DE-PRGs. To stratify the heterogeneity within CD patients, nonnegative matrix factorization clustering was conducted. In terms of immune landscape analysis, the “ssGSEA” method was applied. qRT-PCR was performed to examine the expression levels of the hub DE-PRGs in CD patients and colitis model mice. Ten hub DE-PRGs with satisfactory diagnostic performance were identified and validated: CD44, CIDEC, NDRG1, NUMA1, PEA15, RAG1, S100A8, S100A9, TIMP1 and XBP1. These genes displayed significant associations with certain immune cell types and CD-related genes. We also constructed gene‒microRNA, gene‒transcription factor and drug‒gene interaction networks. CD samples were classified into two PANoptosis patterns according to the expression levels of the hub DE-PRGs. Our results suggest that PANoptosis plays a nonnegligible role in CD by modulating the immune system and interacting with CD-related genes.

## Introduction

Crohn’s disease (CD) is a chronic and progressive inflammatory disease that affects all parts of the gastrointestinal tract and is characterized by segmental, asymmetrical and transmural lesions^1^. CD has constituted a major public health threat since the turn of the twenty-first century. First, CD results in a persistently high disease burden in the Western world, with a prevalence rate surpassing 0.3% in most countries; second, the incidence of CD has been increasing in newly industrialized countries since 1990, such as Brazil, with an annual percentage change of 11.1%^2^.

Several factors are believed to play a role in CD, including environmental factors in genetically susceptible hosts, impaired intestinal barrier function, dysregulated immunity and gut dysbiosis^3^. Excessive cell death has been linked to chronic inflammatory conditions among CD patients. Specifically, overactivation of intestinal epithelial cell apoptosis is commonly observed in CD and is a promising therapeutic target^4,5^. A comparative study showed that necroptosis is active in children with CD and can strengthen the inflammatory process^6^. Aggravation of NLRP3 inflammasome-mediated pyroptosis promotes Crohn’s colitis and gasdermin-E-mediated pyroptosis is involved in CD pathogenesis through the release of proinflammatory cytokines^7,8^. There is evidence at the single-cell transcriptome level that ferroptosis participates in the imbalance of intestinal microenvironment homeostasis in CD patients and inhibition of ferroptosis can reconstruct intestinal barrier^9,10^. Despite these considerable efforts, the mechanism underlying CD is still unclear, and currently available treatments for CD are inadequate.

PANoptosis, a recently established concept, is defined as a form of coordinated programmed cell death that incorporates pivotal features of apoptosis, necroptosis, pyroptosis and ferroptosis and cannot be explained by any of them alone^11,12^. Multiple factors are implicated in inducing PANoptosis, such as infection and self-defects, during which the formation and activation of the PANoptosome complex are essential^13^. Moreover, PANoptosis reportedly plays a part in the pathogenesis and development of numerous illnesses, including cancer and neurodegenerative, metabolic, infectious and inflammatory diseases, such as sepsis, ankylosing spondylitis and CD^13–15^. NINJ1 has been verified to mediate platelet plasma membrane disruption through PANoptosis-related pathways in septic disseminated intravascular coagulation^14^. Previous findings suggest that a potential marker microorganism in ankylosing spondylitis, *Kazachstania pintolopesii*, can trigger the IL-17RA pathway and consequently induce PANoptosis in macrophages^15^. Although prior studies have separately investigated the roles of apoptosis, necroptosis, pyroptosis and ferroptosis in CD, the specific role of PANoptosis in CD etiology remains unknown.

In the first part of this study, we examined the immune landscape of CD and defined differentially expressed PANoptosis-related genes (DE-PRGs) between CD and control samples with microarray-based data from the Gene Expression Omnibus (GEO) database. In the next section, we used machine learning to explore the hub DE-PRGs in CD and further analyzed the interactions of these hub DE-PRGs with immunological characteristics and genes involved in the pathogenesis of CD. We then generated regulatory networks of the hub DE-PRGs. We also identified two distinct PANoptosis patterns (PANclusters) of CD patients and displayed the functional variations between them. Finally, CD samples were obtained and acute 2,4,6-trinitrobenzene sulfonic acid (TNBS)-induced and dextran sodium sulfate (DSS)-induced mouse models were established to validate the identified hub DE-PRGs.

## Methods

### Data sources

Four microarray datasets, GSE95095, GSE100833, GSE75214 and GSE16879, were downloaded from the GEO database (https://www.ncbi.nlm.nih.gov/geo/). To better characterize the changes in the gene expression profiles of CD patients, we analyzed 279 CD samples from involved or inflamed mucosa and 224 control samples from normal mucosa. Additionally, GSE102133 and GSE207022 were included for external validation. We collected PANoptosis-related genes (PRGs) from published articles (Supplementary file 1: Table S1)^16–21^. After removing duplicates, a total of 930 PRGs were included in our study. The gene sets of 28 immune cells possibly associated with CD were obtained from the Molecular Signatures Database (https://www.gsea-msigdb.org/gsea/msigdb/index.jsp). We adopted the search query “Crohn's disease” to obtain information on the top 30 genes involved in CD pathogenesis according to the relevance scores in the GeneCards database (https://www.genecards.org/).

### Principal component analysis and differential gene analysis

Two R packages, “factoextra” and “FactoMineR”, were employed to carry out principal component analysis (PCA), followed by the application of the “ComBat” function in the “sva” package to remove batch effects among distinct datasets^22^. Moreover, the “limma” package was used to screen differentially expressed genes (DEGs) between the CD and control groups, for which the cutoff thresholds were set at |log2 (fold change)| > 1 and adj. *p* < 0.05. We further intersected these DEGs with 930 PRGs through a Venn diagram.

### Enrichment analysis

To determine the potential functions of the genes we identified, we performed Gene Ontology (GO) and Kyoto Encyclopedia of Genes and Genomes (KEGG) analyses via the “clusterProfiler” and “circlize” packages and the “ridgeplot” function in the “DOSE” package of R. We also conducted gene set variation analysis (GSVA) to elucidate the functional variations across different groups.

### Protein–protein interaction network analysis

A protein‒protein interaction (PPI) network was created employing the STRING website (https://cn.string-db.org/) with a confidence threshold of 0.5 for the DEGs acquired from the differential gene analysis. The PPI network was visualized using Cytoscape (version 3.10.0).

### Immune landscape analysis

We used the “ssGSEA” function in the “GSVA” R package to estimate the enrichment scores of 28 specific immune cell genes to reveal the immune landscape of CD. Afterward, Pearson correlation analysis was performed.

### Machine learning

To construct a diagnostic model, we then filtered the DE-PRGs via machine learning. In particular, we defined candidate hub genes based on least absolute shrinkage and selection operator (LASSO) regression, support vector machine (SVM) and random forest (RF) methods, during which the “glmnet”, “kernlab” and “randomForest” R packages were used, respectively. The crossover results of the three techniques were regarded as hub DE-PRGs. Receiver operating characteristic (ROC) curve analysis was subsequently performed to examine the predictive performance of the hub DE-PRGs with the “pROC” package. Meanwhile, an interactive “circos plot” was drawn.

### Construction of regulatory networks

Next, correlation analyses were carried out to outline the crosstalk between immune cells, CD-related genes and hub DE-PRGs. In addition, we constructed gene–microRNA (miRNA), gene–transcription factor (TF) and drug–gene interaction networks of the identified hub DE-PRGs using NetworkAnalyst (https://www.networkanalyst.ca/) and the drug–gene interaction database (https://dgidb.org/).

### Nonnegative matrix factorization

The underlying concept of nonnegative matrix factorization (NMF) may be summarized as follows^23^. The data matrix **X** is represented by ${\mathbb{R}}^{p \times q}$ dimensions, where $p$ represents the data dimension and $q$ represents the sample size. The goal of the NMF approach is to uncover the nonnegative matrices ${\varvec{U}} = \left[ {u_{ij} } \right] \in {\mathbb{R}}^{p \times r}$ and ${\varvec{V}} = \left[ {v_{ij} } \right] \in {\mathbb{R}}^{q \times r}$. In this context, the symbols $u_{ij}$ and $v_{ij}$ denote the specific element located at the $ij$th position in the matrices ${\varvec{U}}$ and ${\varvec{V}}$, respectively. The symbol $r$ denotes the intended reduced dimension. The link between the matrices is estimated as ${\varvec{X}} \approx {\varvec{UV}}^{{\varvec{T}}}$, where ${\varvec{U}}$ is the base matrix and ${\varvec{V}}$ is the coefficient matrix. The calculation of similarity is achieved by determining the distance. The generally used distance metric is the Frobenius norm, which measures the squared Euclidean distance between two matrices^24^. Therefore, the NMF model may be formulated as an optimization problem:

1 $$\mathop {\min }\limits_{{{\varvec{U}},{\varvec{V}}}} \left\| {{\varvec{X}} - {\varvec{UV}}^{{\varvec{T}}} } \right\|_{F}^{2} ,\quad s.t \;\;{\varvec{U}},{\varvec{V}} \ge 0$$

The symbol $\left\| \cdot \right\|_{F}$ denotes the Frobenius norm. The requirements ${\varvec{U}} \ge 0$ and ${\varvec{V}} \ge 0$ imply that every element in matrices ${\varvec{U}}$ and ${\varvec{V}}$ is nonnegative. The objective function of the problem exhibits convexity in either ${\varvec{U}}$ or ${\varvec{V}}$, rendering the identification of the global minimum unattainable. Hence, the following multiplicative updating rules are suggested to obtain the best solutions:

2 $$u_{ij} \leftarrow u_{ij} \frac{{\left( {{\varvec{XV}}} \right)_{ij} }}{{\left( {{\varvec{UV}}^{{\varvec{T}}} {\varvec{V}}} \right)_{ij} }},\;\;\; v_{ij} \leftarrow v_{ij} \frac{{\left( {{\varvec{X}}^{{\varvec{T}}} {\varvec{U}}} \right)_{ij} }}{{\left( {{\varvec{VU}}^{{\varvec{T}}} {\varvec{U}}} \right)_{ij} }}.$$

As an efficient dimensionality reduction tool, NMF is frequently utilized to discover molecular patterns based on high-dimensional genomics data. We used the “NMF” R package to classify 279 CD samples into several clusters based upon the hub DE-PRGs, the geometrical distance between which was visualized by PCA.

### Human subjects

Ten CD patients whose diagnoses of CD were confirmed by their clinical manifestations, endoscopic examinations, and biopsies were recruited. CD samples from the involved mucosa together with control samples from the normal mucosa were acquired during surgery. This protocol was approved by the Ethics Committees of Xiangya Hospital of Central South University. All research was performed in accordance with relevant guidelines and regulations.

### Acute TNBS-induced and DSS-induced mouse models

Twenty-two male C57BL/6J mice aged 6–8 weeks were purchased from Hunan SJA Laboratory Animal Co., Ltd. (Changsha, China) and housed in a specific pathogen-free facility at Central South University. Seven mice were intrarectally administered 100 µL of 2% w/v TNBS (Sigma-Aldrich) while five control mice were intrarectally injected with 100 µL 50% ethanol. All of them were euthanized 7 days later. For the DSS mouse model, to induce acute colitis, five mice received 2% w/v DSS (MP Biomedicals) in drinking water for 7 days and distilled water without DSS for the following 3 days. Throughout the entire experiment, the control group (n = 5) was provided with distilled water. All animals were subsequently euthanized by CO_2_ asphyxiation. All experiments were approved by the Institutional Animal Care and Use Committee of Central South University and reported in accordance with ARRIVE guidelines.

### Quantitative real-time PCR

Total RNA was extracted with TransZol Up reagent (TransGen Biotech), followed by cDNA synthesis with TransScript Uni All-in-One First-Strand cDNA Synthesis SuperMix for qPCR (TransGen Biotech). Quantitative real-time PCR (qRT-PCR) was then performed using *PerfectStart* Green qPCR SuperMix (TransGen Biotech) on an ABI QuantStudio 5 instrument. Gene expression was normalized to that of glyceraldehyde-3-phosphate dehydrogenase. The sequences of the primers used are listed in Supplementary file 2: Table S2.

### Statistical analysis

All analyses were conducted with R software 4.3.0 and Cytoscape software 3.10.0^25^. Unpaired Student's t tests and Wilcoxon rank-sum tests were used to compare the differences between two groups. adj. *p* < 0.05 was considered significant.

**Figure 1:**
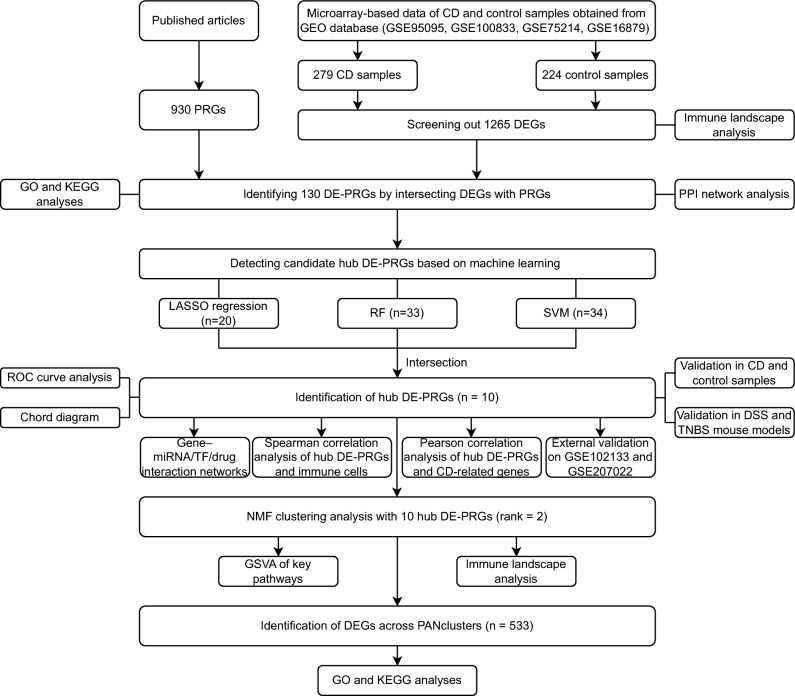
Schematic representation of the study. *PRGs* PANoptosis-related genes, *CD* Crohn’s disease, *GEO* Gene Expression Omnibus, *DEGs* differentially expressed genes, *DE-PRGs* differentially expressed PANoptosis-related genes, *GO* Gene Ontology, *KEGG* Kyoto Encyclopedia of Genes and Genomes, *PPI* protein‒protein interaction, *LASSO* least absolute shrinkage and selection operator, *RF* random forest, *SVM* support vector machine, *ROC* receiver operating characteristic, *DSS* dextran sodium sulfate, *TNBS* 2,4,6-trinitrobenzene sulfonic acid, *miRNA* microRNA, *TF* transcription factor, *NMF* nonnegative matrix factorization, *GSVA* gene set variation analysis, *PANclusters* PANoptosis patterns.

The overall workflow of our study is delineated in Fig. 1.

### Ethics approval and consent to participate

This study was approved by the Ethics Committees of Xiangya Hospital of Central South University and the Institutional Animal Care and Use Committee of Central South University. Informed consent was obtained from all subjects involved in the study.

## Results

### GEO dataset integration and immune landscape of CD

**Figure 2:**
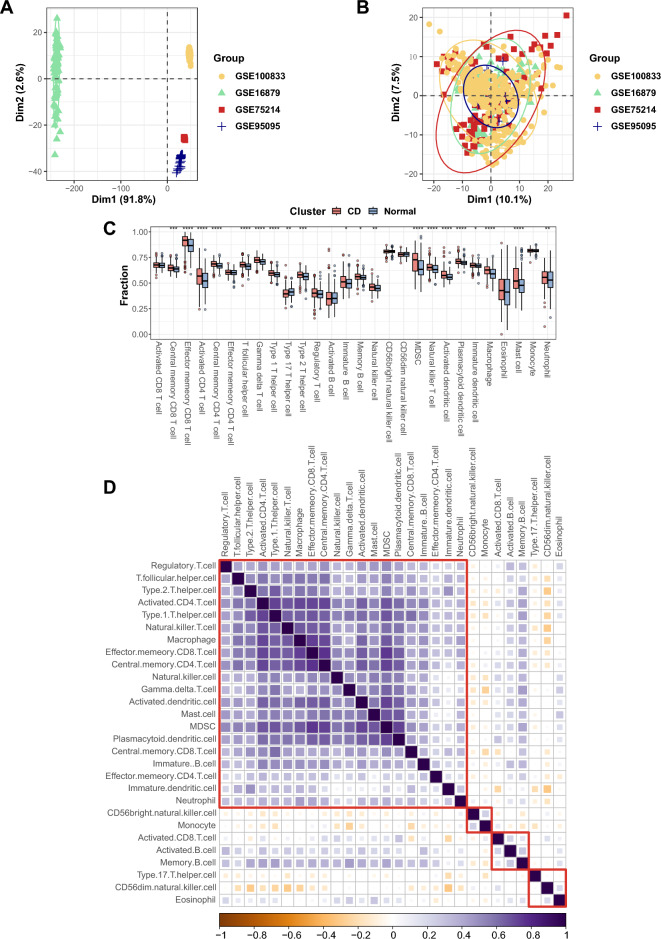
GEO dataset combination and immune landscape of CD. (**A**) PCA between datasets before removal of batch effects. (**B**) PCA between integrated datasets after removal of batch effects. (**C**) Infiltration levels of 28 immune cell subtypes in CD samples and controls. The blue bars represent controls, and the red bars represent CD samples. *p < 0.05; **p < 0.01; ***p < 0.001; ****p < 0.0001. (**D**) Pearson correlation analysis of distinct immune cells. The purple squares represent positive correlations, and the orange squares represent inverse correlations. *GEO* Gene Expression Omnibus, *CD* Crohn’s disease, *PCA* principal component analysis.

We constructed a combined dataset covering 279 CD samples and 224 control samples from mucosa after the removal of batch effects (Fig. 2A,B). A broadly uncoordinated immune response is an indispensable hallmark of CD. With the aim of revealing the immune landscape, we scored the immune cell infiltration of CD patients and controls via the ssGSEA method. As illustrated in Fig. 2C, the infiltration of 20 immune cells in the CD group and control group was significantly different, among which only the scores of T helper 17 (Th17) cells were lower in CD tissues than in control tissues. We then performed a correlation analysis of distinct immune cells, as shown in Fig. 2D. Interestingly, Th17 cells, CD56bright natural killer (NK) cells, CD56dim NK cells and monocytes showed inverse correlations with almost all other immune cells, whereas the other immune cells were generally positively correlated with one another, which deserves special attention.

### Identification of DE-PRGs

**Figure 3:**
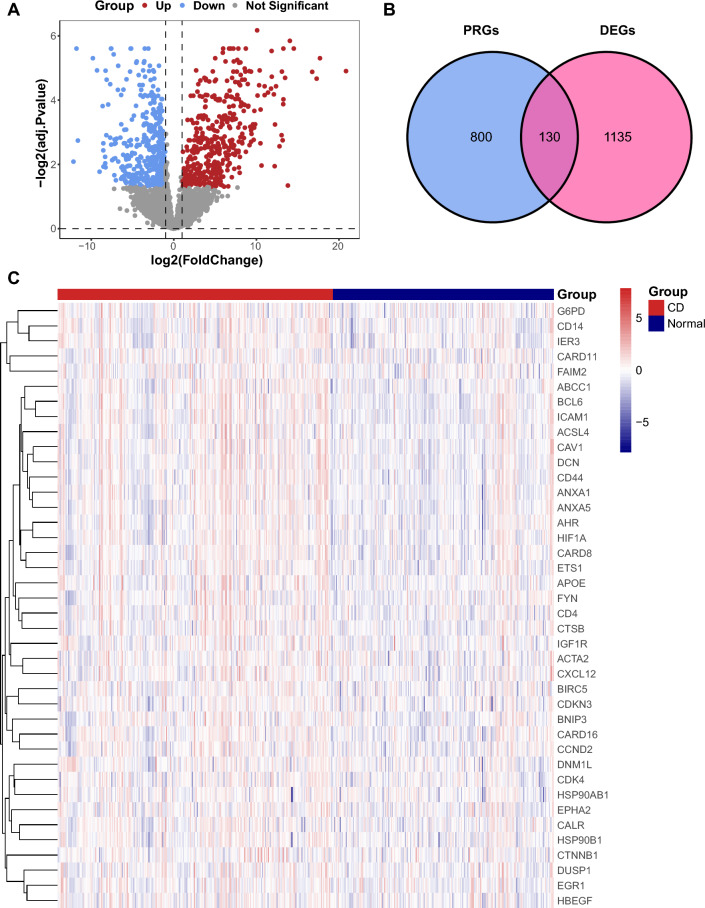
Identification of DE-PRGs. (**A**) Volcano map of the DEGs with the cutoff threshold set at |log2 (fold change)| > 1 and adj. *p* < 0.05. The blue dots represent downregulated DEGs, the red dots represent upregulated DEGs, and the gray dots represent genes with no significant difference. (**B**) Venn diagram of DEGs and PRGs. Pink circle represents DEGs, blue circle represents PRGs, and their overlapping area represents DE-PRGs. (**C**) Clustered heatmap of the top 40 DE-PRGs. Each row represents one of the top 40 DE-PRGs, and each column represents one sample, either CD or normal. *DE-PRGs* differentially expressed PANoptosis-related genes, *DEGs* differentially expressed genes, *PRGs* PANoptosis-related genes, *CD* Crohn’s disease.

A total of 1265 DEGs, consisting of 592 upregulated and 673 downregulated genes, were identified through differential expression analysis (Fig. 3A). A list of possible PRGs was produced from previous research (Supplementary file 1: Table S1). Subsequently, we intersected the 1265 DEGs with 930 PRGs via a Venn diagram; thus, 130 DE-PRGs were identified (Fig. 3B), which were further grouped in a heatmap (Fig. 3C). The overall expression of these DE-PRGs in the CD group and control group is shown in Supplementary file 3: Fig. S1. We could conclude that the vast majority of DE-PRGs were expressed at higher levels in CD tissues than in control tissues.

### Enrichment and PPI network analyses of DE-PRGs

We then examined the latent functions and signaling pathways of the DE-PRGs. GO analysis revealed that these DE-PRGs were predominantly involved in regulation of apoptotic signaling pathway, leukocyte cell‒cell adhesion, regulation of inflammatory response (biological process); membrane raft, membrane microdomain, focal adhesion (cellular component); ubiquitin-like protein ligase binding, ubiquitin protein ligase binding, and phosphatase binding (molecular function) (Supplementary file 4: Fig. S2A). Additionally, DE-PRGs were notably enriched in apoptosis, proteoglycans in cancer, NOD-like receptor signaling pathway, among others, according to the KEGG results (Supplementary file 4: Fig. S2B). Moreover, a PPI network analysis of the DE-PRGs was performed and a complex network of the DE-PRGs was constructed (Supplementary file 5: Fig. S3).

### Identification of hub DE-PRGs

**Figure 4:**
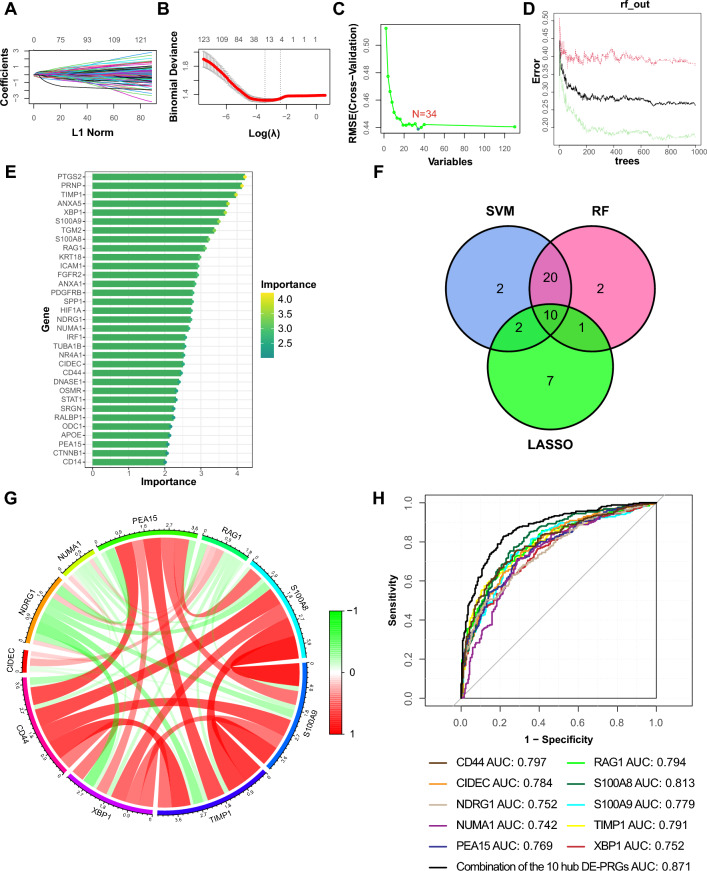
Identification of the hub DE-PRGs. (**A**) Cross-validations of adjusted parameter selection in the LASSO model. Each curve corresponds to one gene. (**B**) LASSO coefficient analysis. Vertical dashed lines are plotted at the best lambda. (**C**) SVM algorithm for hub gene selection. (**D**) Relationship between the number of random forest trees and error rates. (**E**) Ranking of the relative importance of genes. (**F**) Venn diagram showing the 10 hub DE-PRGs identified by LASSO, SVM and RF. Pink circle represents potential hub DE-PRGs identified by RF, blue circle represents potential hub DE-PRGs identified by SVM, green circle represents potential hub DE-PRGs identified by LASSO, and their overlapping area represents the final hub DE-PRGs. (**G**) Chord diagram showing the correlations between the hub DE-PRGs. Red represents positive correlations between different genes and green represents negative correlations between different genes. (**H**) ROC curves of the hub DE-PRGs in CD diagnosis. *DE-PRGs* differentially expressed PANoptosis-related genes, *LASSO* least absolute shrinkage and selection operator, *RF* random forest, *SVM* support vector machine, *ROC* receiver operating characteristic, *AUC* area under the curve, *CD* Crohn’s disease.

To screen the hub DE-PRGs, we first capitalized on three algorithms, LASSO, SVM and RF, and discovered 20, 34 and 33 potential hub DE-PRGs, respectively (Fig. 4A–E). Afterward, 10 hub DE-PRGs were identified through the intersection of the machine learning results, namely CD44, cell death inducing DFFA like effector c (CIDEC), N-myc downstream regulated 1 (NDRG1), nuclear mitotic apparatus protein 1 (NUMA1), proliferation and apoptosis adaptor protein 15 (PEA15), recombination activating 1 (RAG1), S100 calcium binding protein A8 (S100A8), S100 calcium binding protein A9 (S100A9), TIMP metallopeptidase inhibitor 1 (TIMP1) and X-box binding protein 1 (XBP1) (Fig. 4F). Next, we probed their interactions, as shown in Fig. 4G. Most hub DE-PRGs, such as CD44, PEA15, S100A8, S100A9, TIMP1 and XBP1, were closely interrelated. Moreover, NDRG1, NUMA1 and RAG1 generally presented antagonistic effects on the other hub DE-PRGs. Finally, the diagnostic value of each hub DE-PRG in predicting CD was calculated based on our combined dataset (Fig. 4H). All 10 hub DE-PRGs exhibited outstanding predictive performance with area under the curve (AUC) values greater than 0.740. Notably, the AUC reached as high as 0.871 when the 10 hub DE-PRGs were combined (Fig. 4H). In addition, we conducted external validation on the GSE102133 and GSE207022 datasets, respectively. The results were satisfactory, with high AUC values (Supplementary file 6: Fig. S4).

### Relationships between the hub DE-PRGs and immune cells

**Figure 5:**
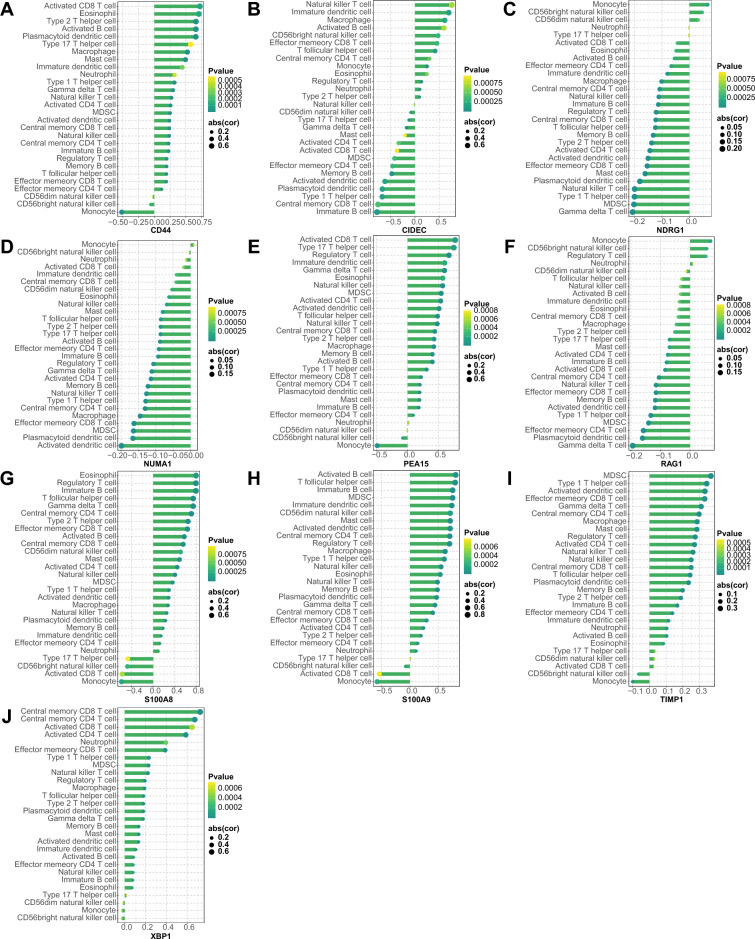
Spearman correlation analysis of hub DE-PRGs with immune cells. The correlations between CD44 (**A**), CIDEC (**B**), NDRG1 (**C**), NUMA1 (**D**), PEA15 (**E**), RAG1 (**F**), S100A8 (**G**), S100A9 (**H**), TIMP1 (**I**) and XBP1 (**J**) gene expressions with immune cells, respectively. The size of the dots represents the strength of gene correlation with immune cells; the larger the dot, the stronger the correlation. The color of the dots represents the p-value; the greener the color, the lower the p-value. *p* < 0.05 was considered statistically significant. *DE-PRGs* differentially expressed PANoptosis-related genes.

Spearman correlation analysis was carried out to determine the interactions between the hub DE-PRGs and immune cells (Fig. 5). CD44, PEA15, S100A8, S100A9, TIMP1 and XBP1 demonstrated noteworthy positive correlations with the infiltration of an abundance of immune cells, except for certain immune cells, such as monocytes and CD56bright NK cells. In contrast, NDRG1, NUMA1, and RAG1 were negatively associated with most types of immune cells, excluding a few immune cells such as monocytes. In addition, the CIDEC fell somewhere between these two extremes.

### Crosstalk between the hub DE-PRGs and CD-related genes

**Figure 6:**
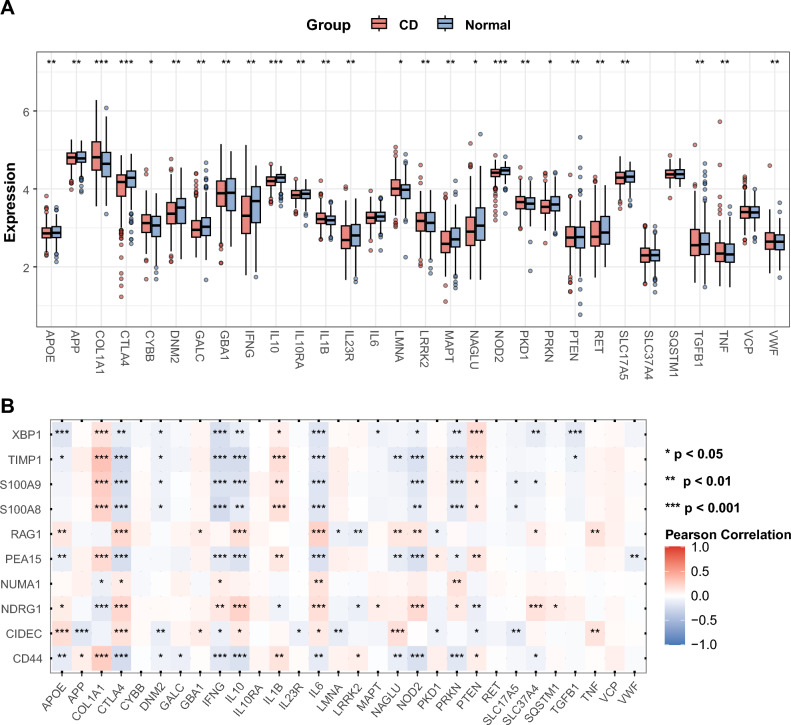
Expression levels of the top 30 CD-related genes and relationships between them and hub DE-PRGs. (**A**) Boxplot of the top 30 crucial genes in relation to CD. The blue bars represent controls, and the red bars represent CD samples. (**B**) Pearson correlation analysis between the top 30 CD-related genes and the 10 hub DE-PRGs. *p < 0.05; **p < 0.01; ***p < 0.001. *CD* Crohn’s disease, *DE-PRGs* differentially expressed PANoptosis-related genes.

The top 30 crucial genes related to CD were extracted from the GeneCards database, and their expression levels were compared between CD samples and normal samples (Fig. 6A). We could easily conclude that a majority of the CD-related genes (26 out of 30) were differentially expressed, especially COL1A1, CTLA4, IL10 and NOD2. Pearson correlation analysis was subsequently conducted to scrutinize the relationships between these CD-related genes and the hub DE-PRGs (Fig. 6B). Notably, CTLA4, one of the most differentially expressed CD-related genes, was significantly associated with each hub DE-PRG. COL1A1, IL10 and NOD2 also presented varying levels of correlation with the hub DE-PRGs. Nevertheless, there were no significant correlations between the hub DE-PRGs and some CD-related genes, including CYBB, IL10RA, RET and VCP.

### Regulatory networks of the hub DE-PRGs

Subsequently, a gene–miRNA interaction network of the 10 hub DE-PRGs consisting of 226 nodes and 338 edges was constructed (Supplementary file 7: Fig. S5 and Supplementary file 8: Table S3). Apparently, miR-124-3p, miR-34a-5p and miR-27a-3p were most strongly associated with the hub DE-PRGs in CD. After that, we generated a gene–TF regulatory network of the 10 hub DE-PRGs (Supplementary file 9: Fig. S6). The 10 hub DE-PRGs were regulated by 35 total TFs. Among them, FOXC1 was found to regulate as many as 7 hub DE-PRGs and S100A8 was regulated by 13 miRNAs (Supplementary file 10: Table S4). In addition, we looked for available drugs that act on the hub DE-PRGs, and a host of drugs were involved (Supplementary file 11: Fig. S7 and Supplementary file 12: Table S5). Specifically, a total of 19 drugs interacted with XBP1, 8 of which inhibited it.

### Recognition of PANclusters

**Figure 7:**
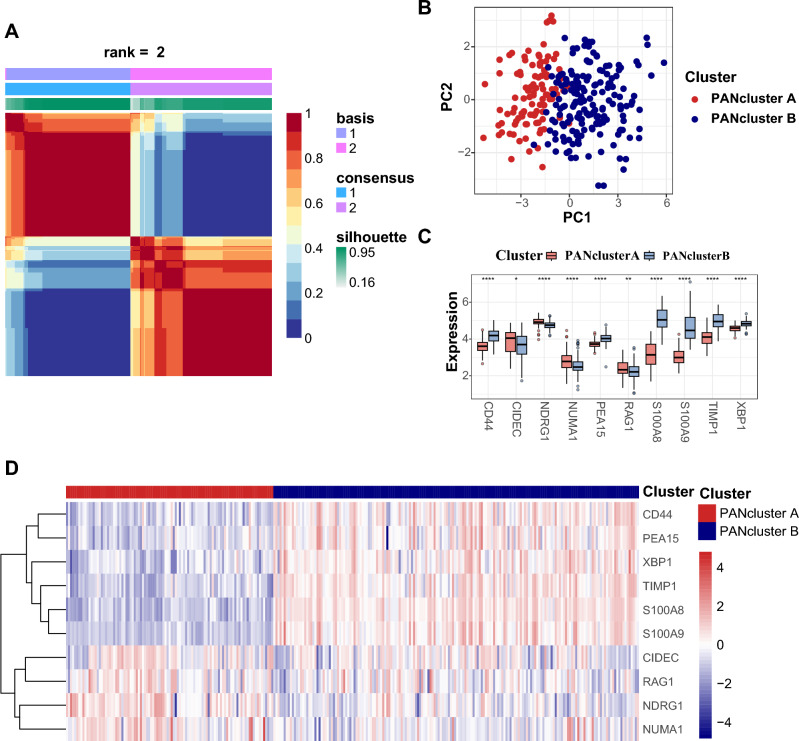
Recognition of PANclusters in CD. (**A**) Unsupervised clustering matrix generated using NMF method when k = 2. (**B**) PCA plot showing the distribution of PANcluster A and PANcluster B. The red dots represent PANcluster A and the blue dots represent PANcluster B. (**C**) Boxplot of the expression levels of the hub DE-PRGs in PANcluster A and PANcluster B. The red bars represent PANcluster A, and the blue bars represent PANcluster B. (**D**) Heatmap of the expression levels of the hub DE-PRGs in PANcluster A and PANcluster B. Each row represents one hub DE-PRG, and each column represents one CD sample. *PANclusters* PANoptosis patterns, *CD* Crohn’s disease, *NMF* nonnegative matrix factorization, *PCA* principal component analysis, *DE-PRGs* differentially expressed PANoptosis-related genes.

To distinguish different PANoptosis patterns in CD patients, we adopted the NMF method for unsupervised clustering on the basis of the 10 hub DE-PRGs. At k = 2, the most stable and optimal PANclusters were identified (Fig. 7A). There were 101 and 178 CD samples in PANcluster A and PANcluster B, respectively. The geometrical distance between the two clusters is shown in Fig. 7B, validating their gene expression heterogeneity. Thereafter, a boxplot and a heatmap were generated to compare the expression levels of the hub DE-PRGs between PANcluster A and PANcluster B (Fig. 7C,D). Specifically, PANcluster A was distinguished by the considerably high expression levels of CIDEC, NDRG1, NUMA1 and RAG1, while the other hub DE-PRGs, that is, CD44, PEA15, S100A8, S100A9, TIMP1 and XBP1, were expressed at higher levels in PANcluster B.

### GSVA of key pathways between the PANclusters

GSVA was performed with the aim of shedding light on the functional diversity patterns of the recognized PANclusters. With regard to Hallmark pathways, increased activity of p53 pathway, androgen response and hypoxia were detected in PANcluster A, whereas mTORC1 signaling, inflammatory response, TNF-α signaling via NF-κB, IL-6/JAK/STAT3 signaling and epithelial mesenchymal transition were increased in PANcluster B (Supplementary file 13: Fig. S8A). In addition, results from the KEGG analysis suggested that PANcluster A had hypoactive ECM–receptor interaction and endocytosis but expressed high levels of genes associated with cytokine–cytokine receptor interaction and numerous signaling pathways, including toll-like receptor signaling pathway and NOD-like receptor signaling pathway (Supplementary file 13: Fig. S8B). Concerning the Reactome-based pathways, PANcluster A showed an increase in the cell cycle pathway, while most pathways, such as cytokine signaling in immune system and extracellular matrix-related pathways, were significantly enriched in PANcluster B (Supplementary file 13: Fig. S8C).

### Characterization of different PANclusters

**Figure 8:**
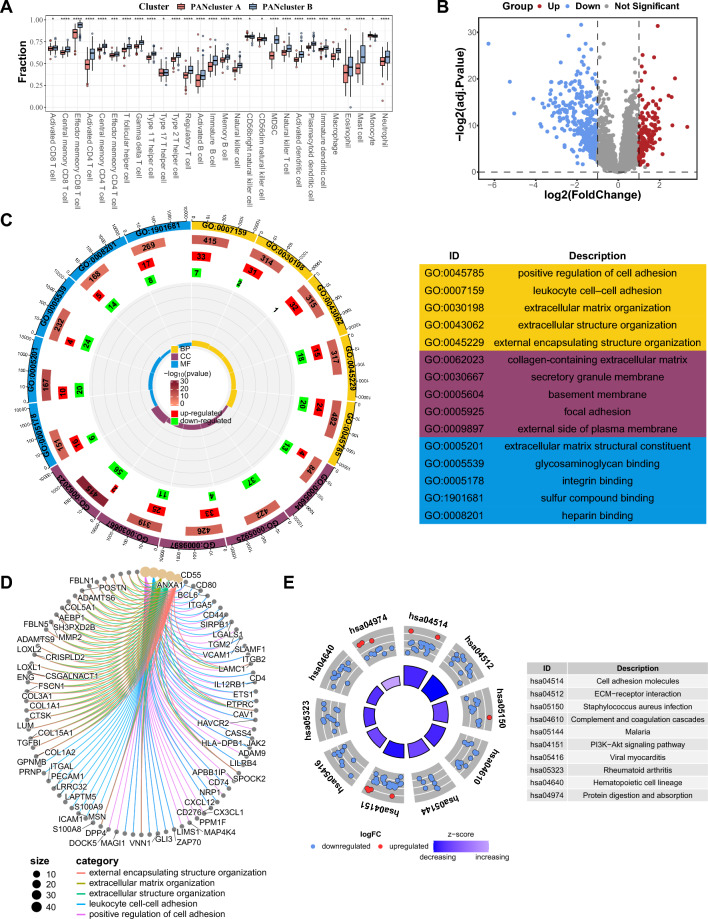
Characterization of different PANclusters. (**A**) Infiltration levels of 28 immune cell subtypes in PANclusters A and B. The red bars represent PANcluster A, and the blue bars represent PANcluster B. (**B**) Volcano map of DEGs between PANclusters A and B. The blue dots represent downregulated DEGs, the red dots represent upregulated DEGs, and the gray dots represent genes with no significant difference. (**C**,**D**) Enriched items in GO analysis based on the DEGs between PANclusters A and B. (**E**) Enriched items in KEGG analysis based on the DEGs between PANclusters A and B. Node color indicates gene expression level; quadrilateral color indicates z-score. *PANclusters* PANoptosis patterns, *DEGs* differentially expressed genes, *BP* biological process, *CC* cellular component, *MF* molecular function, *GO* Gene Ontology, *KEGG* Kyoto Encyclopedia of Genes and Genomes.

To clarify the disparities in the immune system among the PANclusters, we compared their immune microenvironments, as shown in Fig. 8A. Remarkably, the enrichment scores of 26 immune cells were much greater in PANcluster B than in PANcluster A. Consequently, CD56bright NK cells and monocytes were the only two exceptions with higher infiltration degrees in PANcluster A, the explanations behind which demand further investigation. In addition, differential gene analysis revealed 533 DEGs, including 171 upregulated and 362 downregulated genes (Fig. 8B). To learn more about the biological functions and processes linked to these DEGs, GO and KEGG analyses were performed. The 533 DEGs were markedly enriched in the following terms: positive regulation of cell adhesion, leukocyte cell–cell adhesion, and extracellular matrix organization (biological process); collagen-containing extracellular matrix, secretory granule membrane, and basement membrane (cellular component); and extracellular matrix structural constituent, glycosaminoglycan binding, and integrin binding (molecular function) (Fig. 8C,D). Moreover, the 533 DEGs were principally involved in many pathways, such as cell adhesion molecules, ECM–receptor interaction and PI3K-Akt signaling pathway (Fig. 8E).

### Validation of the hub DE-PRGs

**Figure 9:**
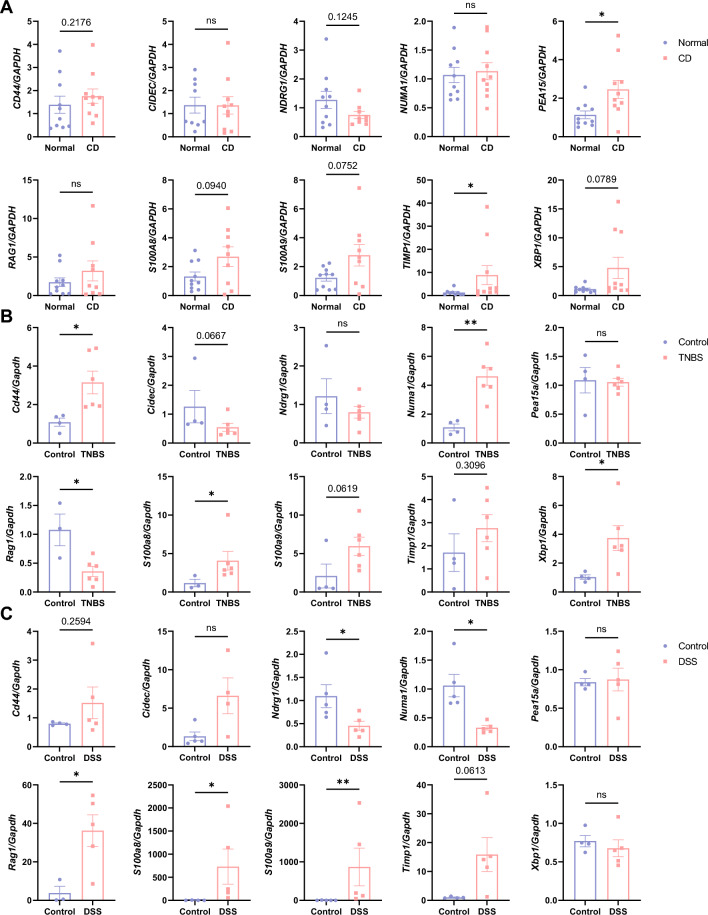
qRT-PCR validation of the hub DE-PRGs in CD patients (**A**), TNBS-induced colitis model (**B**) and DSS-induced colitis model (**C**). The blue dots represent the normal/control tissues, and the red dots represent the diseased tissues. *qRT-PCR* quantitative real-time PCR, *DE-PRGs* differentially expressed PANoptosis-related genes, *CD* Crohn’s disease, *TNBS* 2,4,6-trinitrobenzene sulfonic acid, *DSS* dextran sodium sulfate, *GAPDH* glyceraldehyde-3-phosphate dehydrogenase.

CD and control samples were acquired from 10 patients who were diagnosed with CD, and their demographic and clinical information is presented in Table 1. qRT-PCR was subsequently conducted to determine the relative expression levels of the 10 hub DE-PRGs (Fig. 9A). As expected, the levels of CD44, PEA15, S100A8, S100A9, TIMP1 and XBP1 increased in CD samples compared with those in control samples; while the opposite trend was observed for NDRG1. Moreover, there was no significant difference in the mRNA expression levels of CIDEC, NUMA1 or RAG1. Furthermore, we established classic TNBS and DSS mouse models of CD and collected colon tissues to analyze the expression levels of the hub DE-PRGs in murine colon tissues from the TNBS, DSS and control groups (Fig. 9B,C). Generally, the results of the TNBS model were in line with expectations. Specifically, in TNBS-induced colitis, Cd44, Numa1, S100a8, S100a9, Timp1 and Xbp1 were more highly expressed, while Cidec and Rag1 were less expressed. In addition, the levels of Ndrg1 and Pea15a did not significantly differ between the TNBS group and the control group. Consistent with previous work, in the DSS mouse model, the expression levels of Cd44, S100a8, S100a9 and Timp1 were greater in the mice with colitis; while the expression level of Ndrg1 was lower in the mice with colitis. In addition, no significant difference in the expression levels of Cidec, Pea15a or Xbp1 was detected. Unexpectedly, the expression levels of Numa1 and Rag1 in the DSS group were different from those in the CD and TNBS colitis groups.

**Table 1:** Characteristics of the CD subjects.

	CD patients (n = 10)
Age (median years, IQR)	33 (25)
Female (n, %)	3 (30)
Race (n, %)	
Asian	10 (100)
Disease duration (median years, IQR)	4 (6)
Surgery history (n, %)	10 (100)
Tobacco use (n, %)	
Smoker	4 (40)
Ex-smoker	1 (10)
Nonsmoker	5 (50)
Montreal classification	
Age at diagnosis (n, %)	
Below 16 years (A1)	1 (10)
Between 17 and 40 years (A2)	7 (70)
Above 40 years (A3)	2 (20)
Disease behavior (n, %)	
Nonstricturing, nonpenetrating (B1)	0 (0)
Stricturing (B2)	10 (100)
Penetrating (B3)	3 (30)
Disease location (n, %)	
Ileal (L1)	4 (40)
Colonic (L2)	0 (0)
Ileocolonic (L3)	6 (60)
Perianal disease (n, %)	3 (30)

*CD* Crohn's disease, *IQR* interquartile range.

## Discussion

As a fundamental physiological and pathological process, cell death has a variety of functions, ranging from homeostasis maintenance, embryogenesis and aging to immune system modulation^26^. Dysregulated immune responses and intestinal epithelial cell death, which are common in patients suffering from CD, have long been speculated to contribute to perpetuating inflammation^27^. Existing studies have typically focused on individual forms of cell death rather than the complex and intricate crosstalk among disparate forms of cell death in CD. Currently, a novel form of inflammatory cell death called PANoptosis (apoptosis, necroptosis, pyroptosis and ferroptosis) has been proposed. Herein, we provide a comprehensive and thorough investigation of the roles of PRGs in CD. Our findings highlight the associations between PANoptosis, dysregulation of immunity and CD pathogenesis and development.

Utilizing data from four microarray datasets, we identified 130 DE-PRGs, which were enriched mainly in leukocyte adhesion, inflammatory response, membrane raft and focal adhesion, between the CD group and the normal group. Leukocyte adhesion to the intestinal microvascular endothelium is considered to initiate inflammation in CD^28^. Moreover, lipid rafts have been reported to play a unique role in inflammation and are regarded as a promising therapeutic strategy against inflammation^29^, but their function in CD has not yet been elucidated. Focal adhesion kinase is implicated in the antifibrotic effects of milk fat globule-epidermal growth factor 8 in CD^30^. Likewise, the KEGG enrichment results illustrated that a range of pathways were enriched, such as proteoglycans in cancer and NOD-like receptor signaling pathway. This may partly account for the higher incidence of colorectal cancer in patients with CD^31^. Consistent with earlier reports, NOD2 mutations are likely responsible for the pathogenesis and development of CD^32^, and further investigations are needed to validate the hypothetical underlying mechanisms.

Ten hub DE-PRGs with salient predictive values were further selected and validated by integrated machine learning approaches and experiments: CD44, CIDEC, NDRG1, NUMA1, PEA15, RAG1, S100A8, S100A9, TIMP1 and XBP1. It was not surprising that the expression levels of CIDEC, NUMA1 and RAG1 in human subjects; Ndrg1 and Pea15a in mice with TNBS-induced colitis; and Cidec, Pea15a and Xbp1 in mice with DSS-induced colitis were not significantly different given the small sample sizes. Two plausible explanations for the distinction between the expression levels of NUMA1/Numa1 and RAG1/Rag1 in human and DSS mouse samples are species differences and the limitations of the DSS-colitis model due to inter-batch variability of DSS. Despite the disagreement over the expression of CD44 variants^33^, CD44v7 ligation has been shown to be a trigger for mononuclear cell apoptosis in the lamina propria and hence alleviate the inflammatory immune response among patients with CD^34^. CIDEC interacts with AMPK, a mediator of the mTOR-NF-κB signaling loop, which has provided a promising drug target for CD treatment^35,36^. NDRG1 has been established as a therapeutic candidate for inflammatory bowel disease (IBD)^37^ and may hinder tumor development in an azoxymethane/DSS mouse model^38^. NUMA1 has a substantial influence on the assembly and organization of the mitotic spindle during cell division^39^ and extensive research on this topic has focused on cancer. Nonetheless, the function of NUMA1 in CD has yet to be assessed and reported. PEA15 modulates cellular invasiveness and survival in colorectal carcinoma^40^, but its relevance to CD requires further study. The RAG1 knockout immunodeficient murine model is widely used in IBD-related experiments. S100A8 and S100A9 together form a complex named calprotectin, a verified noninvasive biomarker for IBD that possesses immune-regulatory capabilities^41^. Calprotectin is presumed to engage cytokine receptors and generate reactive oxygen species under constant inflammatory conditions^41^. TIMP1 deficiency can alter inflammation and attenuate fibrosis deterioration in acute and chronic colitis mouse models^42^. The efficacy of infliximab treatment in CD is also likely due to the regulation of TIMP1^43^. Overwhelming endoplasmic reticulum stress might exacerbate intestinal inflammation and XBP1 probably serves as a link that confers genetic risk for CD^44^. Each of the hub DE-PRGs showed significant associations with certain CD-related genes, such as NOD2, TNF and IL6, suggesting that the effects of the hub DE-PRGs on CD strongly depend on their interactions with these CD-related genes.

Gene–miRNA, gene–TF and drug–gene interaction networks were constructed based on the hub DE-PRGs. Altered expression of miR-124-3p has been reported in adult CD patients after exclusive enteral nutrition therapy^45^. Additionally, the miR-124-3p/Annexin 7 pathway has been verified to act downstream of circGMCL1, a CD-associated circRNA, through which NLRP3 inflammasome-mediated epithelial pyroptosis can be alleviated and intestinal barrier function can be protected^46^. Inhibiting miR-34a-5p mitigates intestinal ischemia/reperfusion-induced reactive oxygen species accumulation and apoptosis^47^. Fecal miR-27a-3p is significantly increased in CD^48^. FOXC1 has been acknowledged as a core TF shared by IBD and liver cancer^49^. In summary, all these results provide promising targets for CD but remain to be further studied.

Two PANclusters were detected in the 10 hub DE-PRGs by the NMF algorithm. According to the GO and KEGG analyses, the DEGs between the subclusters were strongly enriched in cell adhesion- and extracellular matrix-related terms. Apart from leukocyte adhesion, the adhesion of various bacteria to intestinal epithelial cells also contributes to the etiology of CD^50,51^. Increased levels of cell adhesion molecules on the surface of immune and intestinal epithelial cells in CD make it possible to deliver drugs to lesions specifically^52^. Extravagant extracellular matrix deposition is a striking hallmark of intestinal fibrosis, a severe complication of CD^53^. Therefore, the susceptibility to intestinal fibrosis is supposed to differ across PANclusters A and B.

Since immune dysregulation is an indispensable feature of CD, we comprehensively explored its immune landscape. Generally, the CD group exhibited a relatively high level of immune cell infiltration, stressing the impacts of abnormal immune responses. Unlike that of other immune cells, the infiltration score of Th17 cells was lower in the CD group than in the control group. Th17 cells are thought to be key effectors of both protective immunity and autoimmune inflammation^54^. Intriguingly, single-cell analyses revealed augmented Th17 activation in CD tissues^55^. One reasonable explanation for this contradiction lies in Th17 plasticity^56^ and in such a context the failure of anti-IL-17 therapy in CD has become understandable and acceptable^54^. There is no denying that additional studies on Th17 cells are necessary. Whether there are significant differences between the CD and normal groups concerning the infiltration of CD56bright NK cells, CD56dim NK cells and monocytes is controversial according to earlier studies^57–59^. Our results support the view that the infiltration of CD56bright NK cells, CD56dim NK cells and monocytes does not differ between CD and normal samples, all of which are negatively linked to most other immune cells. This divergence may be attributed to heterogeneity among CD patients and distinct datasets and algorithms. More samples and more advanced algorithms are indispensable to end this controversy.

To the best of our knowledge, our study is the first to comprehensively describe the roles of PANoptosis in CD. Not only have the roles of PRGs been evaluated, but the interactions among PANoptosis, aberrant immunity and CD pathogenesis have also received particular attention. In addition, the combined analysis of three advanced machine learning techniques effectively reduced bias. Another key advantage lies in that we innovatively categorized CD patients based on hub DE-PRGs. Specific or even personalized diagnosis and therapy may be inspired by our findings. Finally, we not only recruited human subjects but also established TNBS-induced and DSS-induced mouse models to validate our results.

It cannot be denied that several limitations still exist in our study. First, limited by the raw data, we were incapable of tracing the specific sampling locations of the human tissues for the generation of the microarray datasets and connecting expression profiles with specific phenotypes. Second, the sample sizes of both human subjects and mice were small and we established only two acute mouse models to confirm our results. Accordingly, we are going to enlarge the sample size next and employ chronic murine models of CD. To further elucidate the mechanisms underlying the impacts of hub DE-PRGs on CD, we also plan to carry out causal inference analysis.

## Conclusions

In summary, we screened and validated 10 hub DE-PRGs in CD patients via integrated bioinformatics, machine learning and experiments, all of which performed well in terms of CD diagnosis. Each hub DE-PRG showed significant associations with certain immune cells and CD-related genes, indicating that their roles in CD involved modulating the immune system and interacting with genes involved in the pathogenesis of CD. Gene–miRNA, gene–TF and drug–gene interaction networks were subsequently generated based on the hub DE-PRGs, which could provide directions for subsequent CD therapies. We also identified two PANclusters with distinct immune infiltration and functional patterns. Undoubtedly, further studies are urgently warranted to uncover the involvement of PANoptosis in CD etiology and develop more effective diagnostic and therapeutic strategies for CD.

### Supplementary Information

Supplementary Information.

## Data Availability

The datasets supporting the conclusions of this article are available in the [Gene Expression Omnibus database] repository, [https://www.ncbi.nlm.nih.gov/geo/]. The GEO accessions are [GSE95095], [GSE100833], [GSE75214], [GSE16879], [GSE102133] and [GSE207022].

## Abbreviations

AUC: Area under the curve
CD: Crohn’s disease
DEG: Differentially expressed gene
DE-PRGs: Differentially expressed PANoptosis-related genes
DSS: Dextran sodium sulfate
GEO: Gene Expression Omnibus
GO: Gene Ontology
GSVA: Gene set variation analysis
IBD: Inflammatory bowel disease
KEGG: Kyoto Encyclopedia of Genes and Genomes
LASSO: Least absolute shrinkage and selection operator
miRNA: MicroRNA
NK: Natural killer
NMF: Nonnegative matrix factorization
PANcluster: PANoptosis pattern
PCA: Principal component analysis
PPI: Protein‒protein interaction
PRGs: PANoptosis-related genes
qRT-PCR: Quantitative real-time PCR
RF: Random forest
ROC: Receiver operating characteristic
SVM: Support vector machine
TF: Transcription factor
Th17: T helper 17
TNBS: 2,4,6-Trinitrobenzene sulfonic acid
