# Supplementary material for: Characterization of PANoptosis-related genes in Crohn’s disease by integrated bioinformatics, machine learning and experiments
Source: Sci Rep. 2024 May 22;14:11731. doi: 10.1038/s41598-024-62259-w (PMC11111690; doi:10.1038/s41598-024-62259-w)
Supplement: Supplementary file 1 — Supplementary Information. [file 41598_2024_62259_MOESM1_ESM.zip › Supplementary files.docx]

**Characterization of PANoptosis-related genes in Crohn's disease** **by integrated bioinformatics, machine learning and experiments**

Yang Yang^1,2,3,4^, Alphonse Houssou Hounye^5^, Yiqian Chen^1,2,3^, Zhuqing Liu^1,2,3,4^, Guanzhong Shi^3,4^, Ying Xiao^1,2,3^*

^1^Department of Gastroenterology, Xiangya Hospital, Central South University, Changsha, Hunan, China

^2^Hunan International Scientific and Technological Cooperation Base of Artificial Intelligence Computer Aided Diagnosis and Treatment for Digestive Disease, Changsha, Hunan, China

^3^National Clinical Research Center for Geriatric Disorders, Xiangya Hospital, Central South University, Changsha, Hunan, China

^4^Xiangya School of Medicine, Central South University, Changsha, Hunan, China

^5^School of Mathematics and Statistics, Central South University, Changsha 410008, China

*Corresponding author:

Ying Xiao. Department of Gastroenterology, Xiangya Hospital, Central South University, Changsha, Hunan, China. Tel: +86-147-89743529. Email: xiaoying1113@csu.edu.cn.

**Legends for supplementary tables**

**Supplementary file 1: Table S1.** PRGs reported in published articles. PRGs, PANoptosis-related genes.

**Supplementary file 2: Table S2.** Primer sequences of qRT-PCR. qRT-PCR, quantitative real-time PCR.

**Supplementary file 8: Table S3.** List of miRNAs interacting with the hub DE-PRGs. miRNA, microRNA. DE-PRGs, differentially expressed PANoptosis-related genes.

**Supplementary file 10: Table S4.** List of TFs regulating the hub DE-PRGs. TFs, transcription factors. DE-PRGs, differentially expressed PANoptosis-related genes.

**Supplementary file 12: Table S5.** List of drugs acting on hub DE-PRGs. DE-PRGs, differentially expressed PANoptosis-related genes.

**Supplementary figures**

**
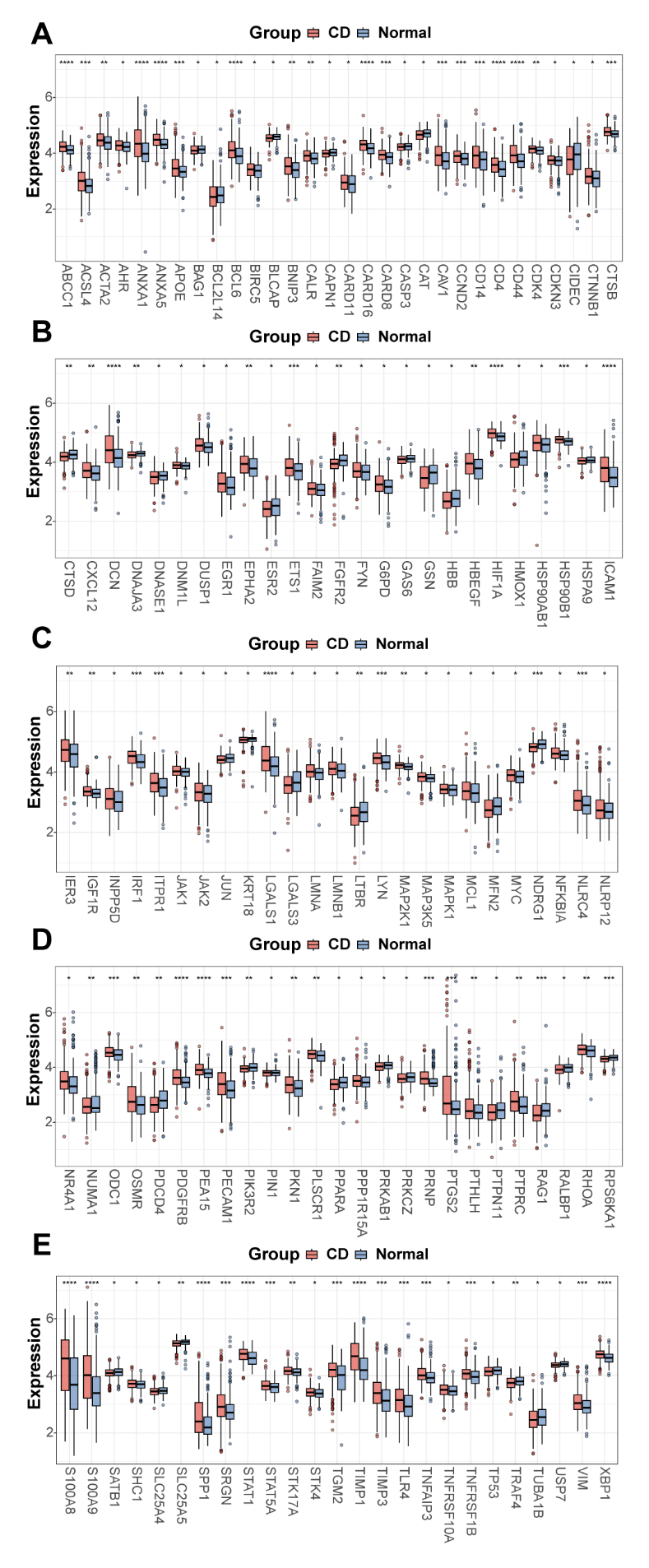
**

**Supplementary file 3: Figure S1.** Boxplots of the expression of 130 DE-PRGs in the CD group and control group. The blue bars represent controls, and the red bars represent CD samples. **p* < 0.05; ***p* < 0.01; ****p* < 0.001; *****p* < 0.0001. DE-PRGs, differentially expressed PANoptosis-related genes. CD, Crohn’s disease.


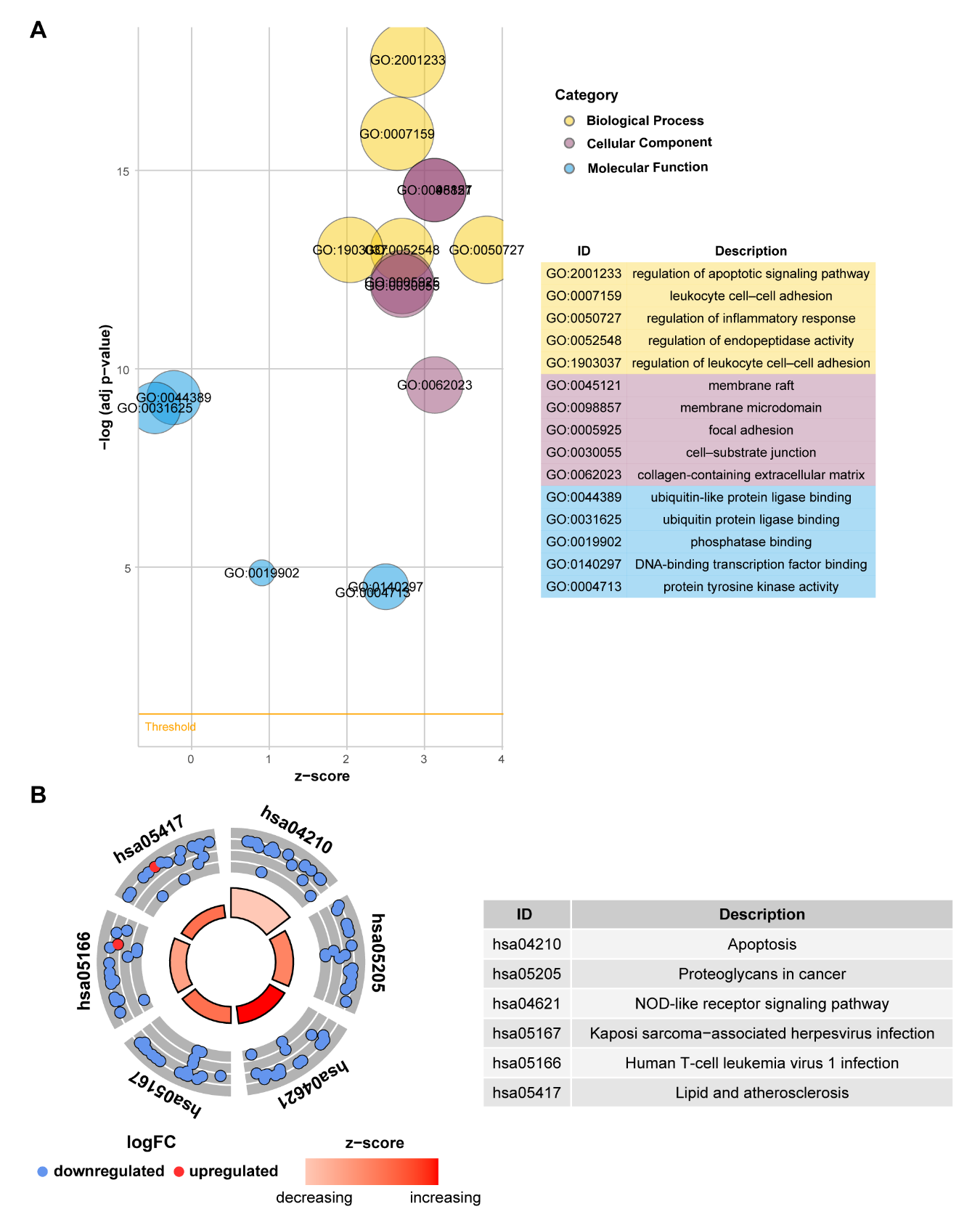


**Supplementary file 4: Figure S2.** Functional and signaling pathway enrichment analyses of the DE-PRGs. (A) Enriched items in GO analysis. The vertical coordinate is the significance of the enrichment result; the horizontal coordinate is the z-score; node color indicates biological process, cellular component and molecular function; node size indicates the number of genes contained in the current GO term. (B) Enriched items in KEGG pathway analysis. Node color indicates gene expression level; quadrilateral color indicates z-score. DE-PRGs, differentially expressed PANoptosis-related genes. GO, Gene Ontology. KEGG, Kyoto Encyclopedia of Genes and Genomes.


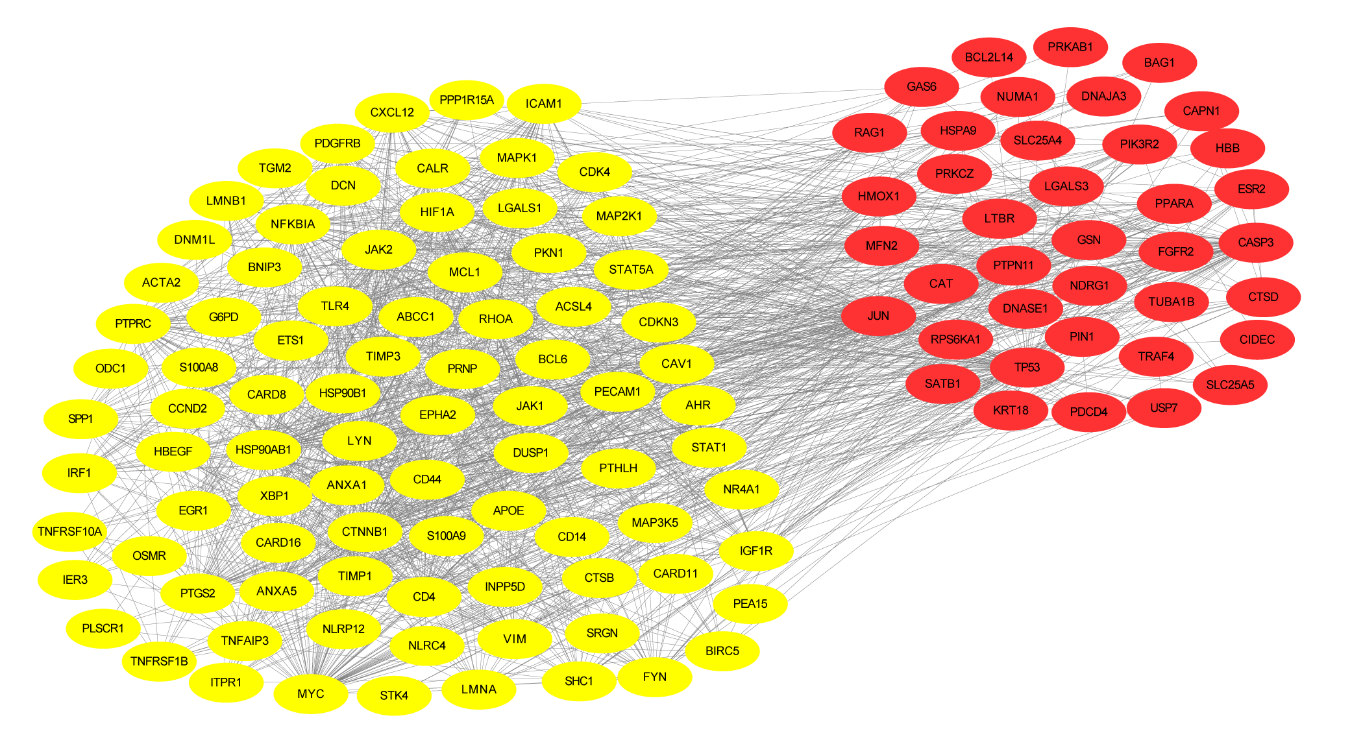


**Supplementary file 5: Figure S3.** A PPI network of all the DE-PRGs. The yellow and red circles represent upregulated and downregulated DE-PRGs, respectively. PPI, protein‒protein interaction. DE-PRGs, differentially expressed PANoptosis-related genes.


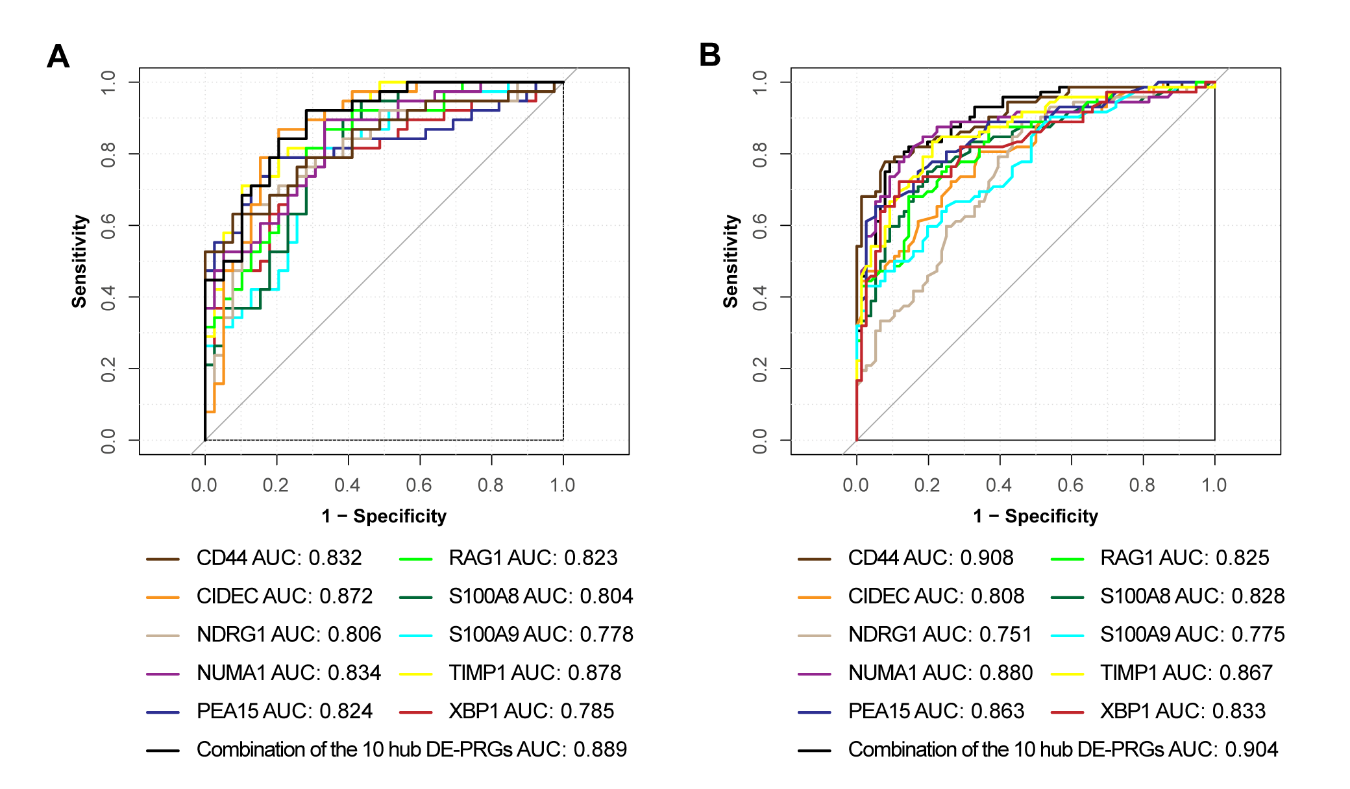


**Supplementary file 6: Figure S4.** External validation of the diagnostic value of the hub DE-PRG in predicting CD on GSE102133 (A) and GSE207022 (B), respectively. DE-PRGs, differentially expressed PANoptosis-related genes. CD, Crohn’s disease. AUC, area under the curve.


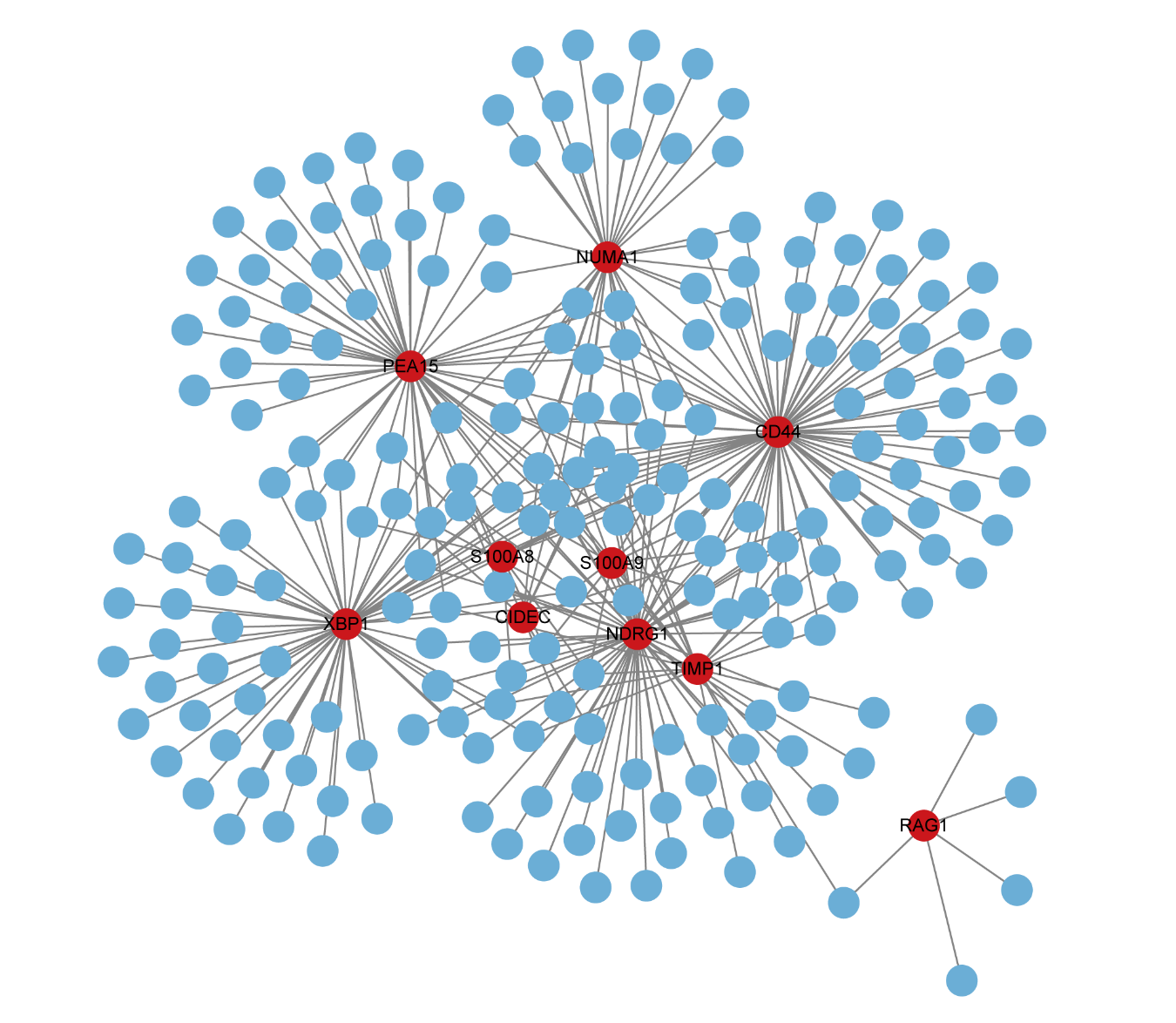


**Supplementary file 7: Figure S5.** Integrated gene–miRNA networks for the hub DE-PRGs. The red circles represent the hub DE-PRGs, and the blue circles represent miRNAs. miRNA, microRNA. DE-PRGs, differentially expressed PANoptosis-related genes.


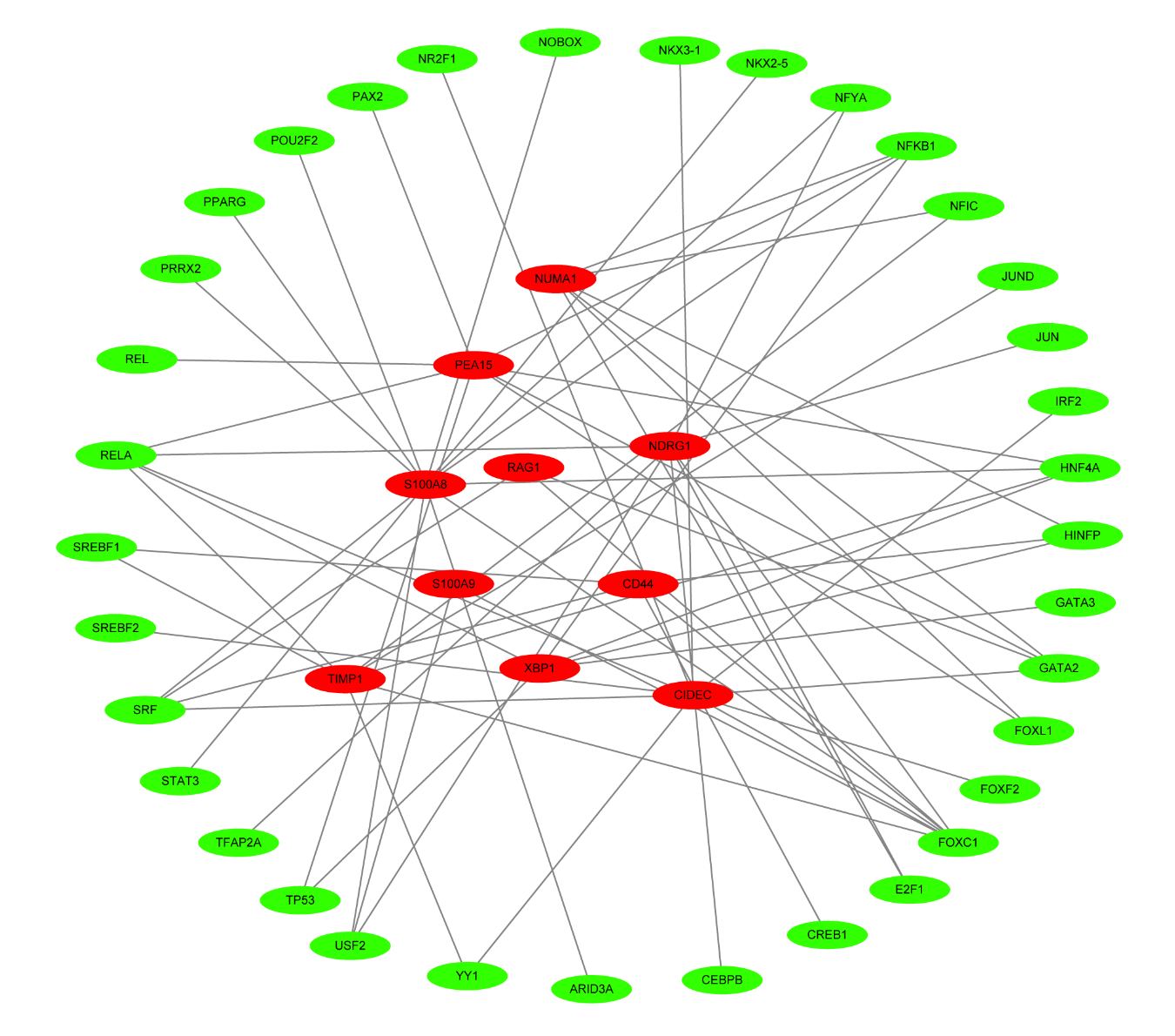


**Supplementary file 9: Figure S6.** Integrated gene–TF networks of the hub DE-PRGs. The red circles represent the hub DE-PRGs, while the green circles represent TFs. TF, transcription factor. DE-PRGs, differentially expressed PANoptosis-related genes.


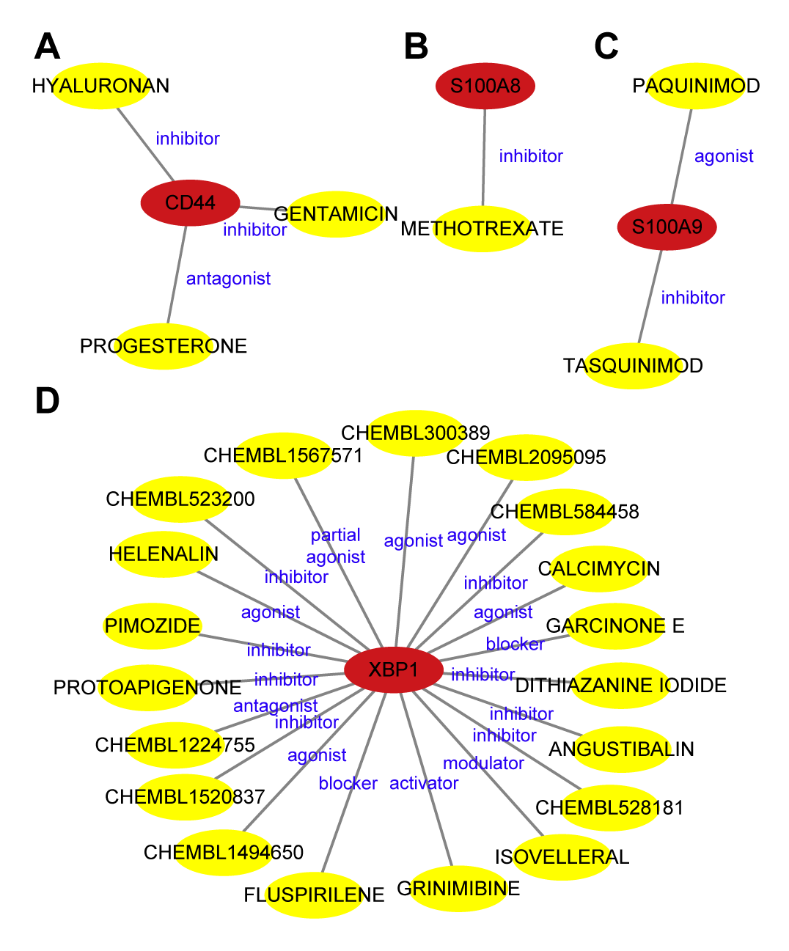


**Supplementary file 11: Figure S7.** Drug-gene interaction networks of the hub DE-PRGs. The red circles represent the hub DE-PRGs, while the yellow circles represent drugs. DE-PRGs, differentially expressed PANoptosis-related genes.

**
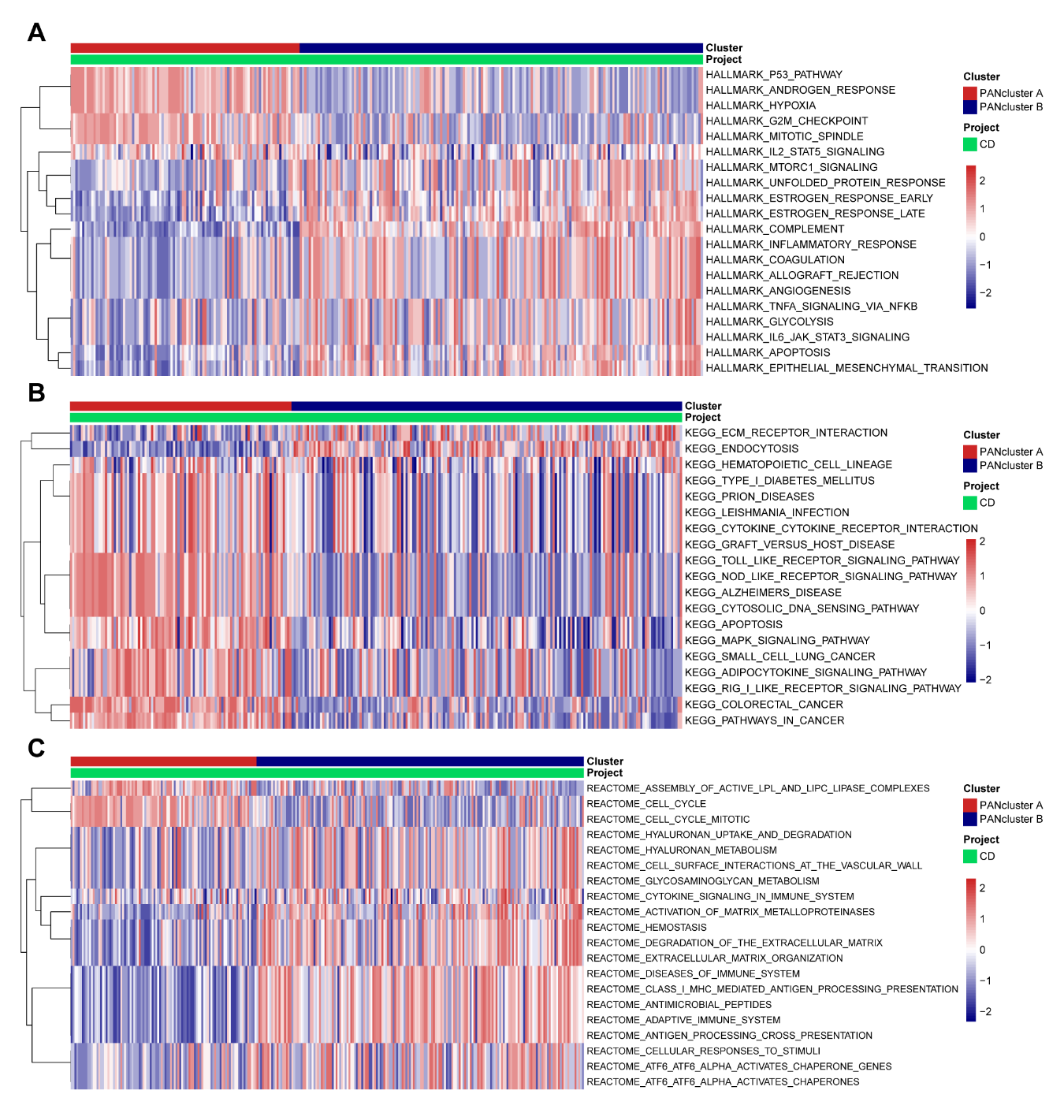
**

**Supplementary file 13: Figure S8.** GSVA of key pathways between PANclusters. (A) Enriched pathways based on the Hallmark pathway. (B) Enriched pathways based on the KEGG pathway. (C) Enriched pathways based on the Reactome pathway. Each row represents an enriched pathway, and each column represents a CD sample. GSVA, gene set variation analysis. PANclusters, PANoptosis patterns. CD, Crohn’s disease. KEGG, Kyoto Encyclopedia of Genes and Genomes.
